# Nutrient Enrichment Alters Phenotypic Selection on Plant Traits in an Annual Herb on the Tibetan Plateau

**DOI:** 10.1002/ece3.71592

**Published:** 2025-06-14

**Authors:** Lu Ningna, Hou Meng, Ma Yan, Øystein H. Opedal, Zhao Zhigang

**Affiliations:** ^1^ School of Life Science North‐West Normal University Lanzhou China; ^2^ College of Ecology Lanzhou University Lanzhou China; ^3^ College of Grassland Agriculture Northwest A&F University Yangling China; ^4^ Department of Biology Lund University Lund Sweden

**Keywords:** alpine meadow, floral traits, nutrient addition, *Pedicularis szetschuanica*, phenotypic selection

## Abstract

Nutrient enrichment is an increasingly important consequence of anthropogenic activities. Nutrient enrichment can alter the composition, diversity, and functioning of terrestrial plant communities, yet its effect on evolutionary processes in plant populations has been less well studied. To understand the evolutionary consequence of long‐term soil nutrient enrichment, we examine the effects of nutrient addition (N or P) on plant traits, female reproductive success, and pattern of phenotypic selection in the annual plant *Pedicularis szetschuanica* M. in an alpine meadow on the Tibetan Plateau. Nutrient addition generally increased plant height and reduced tube length and nectar production per flower. Surprisingly, the effects of N and P addition on seed number per plant were reversed between years. Despite variation in traits, mean fitness, and opportunity for selection among nutrient treatments, patterns of selection changed only for nectar production, where we detected N‐mediated selection favoring greater nectar production. This study suggests that nutrient enrichment can alter patterns of phenotypic selection, potentially influencing the evolution of floral traits even if pollinators play a limited role in selection.

## Graphical abstract

This study examined the effects of nutrient addition on plant traits, female reproductive success, and patterns of phenotypic selection in the annual plant *Pedicularis szetschuanica* M. in alpine meadow on the Tibetan Plateau. The results suggest that nutrient enrichment can alter patterns of phenotypic selection, potentially influencing the evolution of floral traits even if pollinators play a limited role in selection.

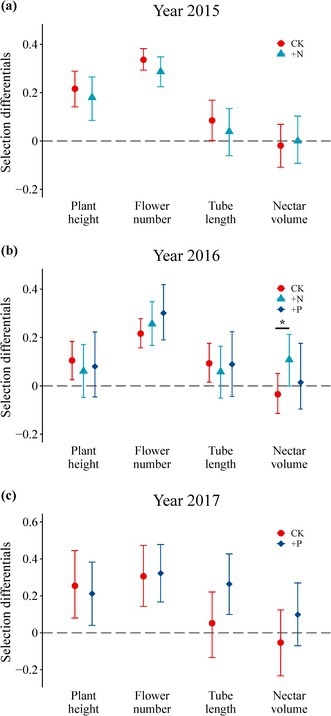

## 1 Introduction

Nutrient enrichment is an increasingly important consequence of anthropogenic activities (Harpole et al. 2011). Nitrogen (N) and phosphorus (P), the most limiting nutrients for primary production in terrestrial ecosystems (Elser et al. 2007; Harpole et al. 2011; Hou et al. 2020), influence plant growth, reproduction (e.g., Campbell and Halama 1993; Burkle and Irwin 2010; Fortier and Wright 2021), and species interactions (e.g., Wallace et al. 1997; David et al. 2019; Carvalheiro et al. 2021). Since the industrial revolution, human activities have amplified global N and P cycles by *c*. 100% and *c*. 400%, respectively (Falkowski et al. 2000). Through its effects on individual plant species, nutrient enrichment can alter the composition, diversity, and functioning of terrestrial plant communities (Clark and Tilman 2008; Borer et al. 2014). In contrast to the focus on community‐level ecological effects of nutrient enrichment (e.g., Hautier et al. 2009; Eskelinen et al. 2022; Cheng et al. 2023), its effect on evolutionary processes in plant populations has been less well studied. For plant species that are adapted to comparatively nutrient‐poor soils, chronic nutrient enrichment could cause evolutionary change by modifying patterns of phenotypic selection (Barnosky 2001; MacColl 2011).

Floral traits are hypothesized to evolve primarily in response to pollinator‐mediated natural selection (Darwin 1862; Faegri and van der Pijl 1966; Fenster et al. 2004; Harder and Johnson 2009). However, a recent meta‐analysis suggests that selection on floral traits mediated by abiotic factors (e.g., soil water and nutrients) can be as strong or stronger than selection mediated by pollinators (Caruso et al. 2020). Furthermore, pollinator‐mediated selection on floral traits can be altered by resource availability (Wu et al. 2022, 2023). These observations strongly suggest that abiotic resources (e.g., N and P) also play an important role in the evolution of floral traits (Galen 1999; Strauss and Whittall 2006). Empirical studies addressing these effects are lacking (MacColl 2011; Caruso et al. 2017). Nutrient enrichment experiments provide a powerful approach to examine the evolutionary consequences of eutrophication by quantifying the relationship between traits and fitness at natural vs. increased nutrient levels, which can in turn facilitate the identification of causal selective agents (Wade and Kalisz 1990).

Resource supplementation can influence natural selection on plant traits by altering the opportunity of selection or the functional relationships between traits and fitness. Plant sexual reproduction (seed production) is often limited by both resource availability and pollination services (Haig and Westoby 1988; reviewed in Ashman et al. 2004). Variation in plant size, floral advertisements, and rewards is often associated with resource availability (e.g., Burkle and Irwin 2009; Carvalheiro et al. 2021). Larger plants and enhanced floral advertisements (e.g., flower number, flower size) and reward traits (e.g., nectar production) resulting from greater nutrient supply are expected to increase pollinator attraction because these traits often influence pollinator visitation (e.g., Galen et al. 1985; Galen et al. 1987; Schemske and Horvitz 1988). Thus, nutrient supply can indirectly increase seed output if plants are pollen‐limited under ambient nutrient conditions. For example, nitrogen addition had a strong positive effect on seed production of the alpine shrub *Chuquiraga oppositifolia*, and this effect was indirect through an effect of larger floral displays on pollinator visits (Munoz et al. 2005). In these situations, we expect nutrient addition to lead to intensified selection on flower traits. Because nitrogen addition is expected to intensify size‐asymmetric aboveground competition for light (i.e., taller individuals receive disproportionately more light per unit size and sustain more seeds; Weiner 1990; Schwinning and Weiner 1998; Hautier et al. 2009), we specifically expect selection to favor taller plants after nutrient addition. These examples illustrate how resource addition could mediate selection by changing the functional relationships between resource‐related traits and female fitness.

An alternative effect of resource addition is a homogeneous increase in trait values and fitness across individuals. In this case, nutrient addition may have no or minor effects on phenotypic selection. However, because the opportunity for selection is determined by the variance in relative fitness (Crow 1958), selection should be stronger in environments where mean fitness is relatively low, which is usually associated with greater variance in relative fitness (Arnold and Wade 1984; Rundle and Vamosi 1996; Caruso et al. 2017). Resource supplementation could thus affect patterns of selection by reducing the opportunity for selection. Such research is helpful to understand how plant species adapt to environments enriched with nutrients due to human disturbances.

However, we are not aware of any study that has assessed experimentally how soil nutrient addition affects the strength and patterns of natural selection on floral traits in natural communities. Here, we leverage a long‐term nutrient addition experiment to examine the effects of soil nutrient addition (with N or P) on plant traits, female reproductive success, and phenotypic selection in the annual *Pedicularis szetschuanica* M. in the alpine meadow of the Tibetan Plateau. Given the dependence of plant growth and reproduction on soil nutrient availability in alpine ecosystems, we hypothesized that N and P addition should increase plant size (plant height), floral traits (flower number, flower size, and nectar production), and seed number of *P. szetschuanica*. Because increased mean seed number per plant in the nutrient treatments may reduce the opportunity for selection, we expect weaker selection after nutrient addition. Furthermore, we expect a negative relationship between selection intensity and opportunity for selection across treatments. Finally, if traits that grow larger under nutrient supplementation enhance attractiveness to pollinators, nutrient addition should lead to stronger selection.

## 2 Materials and Methods

### 2.1 Study Site and Plant Species

The experiment was carried out at the Gansu Gannan Grassland Ecosystem National Observation and Research Station of Lanzhou University (Maqu branch) in the eastern Tibetan Plateau (33°40′5″ N, 101°51′44″ E, altitude 3500 m a.s.l.), Gansu, China. The vegetation at the study site was a typical alpine meadow, dominated by graminoids, including *Kobresia capillifolia*, *Agrostis hugoniana*, *Poa poophagorum*, and *Elymus nutans*. The study area had a prolonged frost period (> 270 days per year) and a short flowering season (mainly from June to August).

Our study species, *Pedicularis szetschuanica* M. (Orobanchaceae), is an annual herb, widely distributed in alpine grasslands (3300–4500 m) of the Qinghai‐Tibetan Plateau. Individual plants usually produce one to several spicate inflorescences, which open sequentially from bottom to top (acropetally). The flowers are hermaphrodite, and the corolla tube is purple to red and longer than the galea. The nectary is at the base of the floral tube. Although most of the *Pedicularis* species are usually pollinated by bumble‐bees (Macior 1982), the flowering plants of *P. szetschuanica* M. are visited and pollinated by flies, bees, beetles, and ants on the eastern Qinghai‐Tibetan Plateau. Plants bloom from June through August, with each flower lasting 4–8 days.

### 2.2 Experimental Design

The experiment comprised three treatments and was established at the study site since May 2011, using a random‐block experimental design. The treatments included nitrogen addition (+N), phosphorus addition (+P), and a control treatment without nutrient addition (C). We replicated each treatment six times. The area of each plot was 10 × 20 m, and plots were separated by 1 m buffer strips. The nutrient‐addition treatments were implemented by fertilizing with NH_4_NO_3_ (5 g N m^−2^ per year) and Ca(H_2_PO_4_)_2_ (8 g P m^−2^ per year), respectively, applied before a rain event in early May each year since 2011. The study site was fenced during the growing season but grazed by livestock over winter.

### 2.3 Plant Sampling and Trait Measurements

The data analyzed here were collected in three consecutive years from 2015 to 2017. Because the abundance of *Pedicularis szetschuanica* varied substantially across plots, treatments, and years, we focused on the N‐addition and control plots in 2015 and 2016, and the P‐addition and control plots in 2016 and 2017. From early July to early August, during the peak flowering period of *P. szetschuanica*, we randomly tagged 80 individuals in the control plots and 60 individuals in the N‐addition plots in 2015, 60 individuals each from the control, +N, and +P plots in 2016, and 70 individuals each from the control and +P plots in 2017.

For each tagged plant, we measured plant height from the ground to the topmost open flower using a steel ruler, counted the total number of flowers per plant, measured tube length using digital calipers (Mahr Federal 16 ER Digital Caliper; Germany), and nectar production as the length of the liquid column extracted with a 0.5 μL capillary tube. The first three flowers at the bottom of the spike per plant were chosen for the measurements when one‐third of the flowers per inflorescence had bloomed. We extracted nectar from the base of the floral tube at 08.00 and 10.00 am in the field on a sunny day. The flowers used for nectar measurements were bagged 1 day before nectar collection to prevent pollinators from affecting the nectar measurement. The nectar volume was calculated as the length of the liquid column in the capillary tube divided by the total length of the capillary tube and multiplied by the total volume of the capillary tube (Cruden and Hermann 1983).

To quantify the composition of the pollinator assemblage, we chose 30 plants per treatment, for which we recorded pollinator visits using camcorders (Sony HDR) on tripods. These observations were made between 10:00 am and 04:30 pm on sunny, windless days. Each individual plant was recorded three times for 15 min with 30–60 min breaks between intervals. In the videos, pollinator visits were recorded whenever insects contacted floral reproductive organs. Flower visitors were identified at the species or morphospecies level by referring to previously collected insect specimens from the study area (Gao et al. 2021). We estimated the visitation rate of each plant individual as the number of pollinator visits observed per hour. When the fruits matured, all fruits on each plant were collected, and for six of them, we counted the number of seeds and unfertilized ovules. Lifetime female reproductive fitness was estimated for each plant by multiplying the mean seed number in the six fruits by the total number of fruits produced by that plant.

### 2.4 Statistical Analyses

All statistical analyses were performed in R version 4.3.0 (R Core Team 2023). We used linear mixed models to examine the effects of nutrient treatment (N and P addition), year, and their interaction on phenotypic traits, visitation rate, and seed number per plant. We examined the relationship between phenotypic traits and pollinator visitation rate through linear regression analysis within each treatment.

We estimated selection differentials and selection gradients following the methods of Lande and Arnold (1983). For each treatment, we calculated relative female fitness by dividing the estimated seed number of each individual by the mean estimated seed number in the population. We then quantified the opportunity for selection (I) through each fitness measure as the variance in relative fitness (Arnold and Wade 1984) and derived 95% confidence intervals through non‐parametric bootstrapping using the BOOT R package (Davison and Hinkley 1997; Canty and Ripley 2022). All traits were standardized to zero mean and unit variance before fitting the models in each treatment, thus yielding variance‐standardized selection gradients (Hereford et al. 2004; Opedal 2021). We estimated selection differentials as the regression slope of relative fitness on individual traits and multivariate selection gradients as the partial slopes from multiple‐regression models with relative female fitness as the response variable and variance‐standardized trait values as explanatory variables. The variance inflation factors (VIF) were < 2 in all models, suggesting no serious multicollinearity. We determined *p*‐values and 95% bias‐corrected and accelerated confidence intervals using a bootstrap method (Efron and Tibshirani 1993; Fox and Weisberg 2019; Hou et al. 2024).

To assess whether the nutrient‐addition treatments influenced patterns of selection, we fitted ANCOVA models with relative fitness as the response variable, the four standardized traits (plant height, number of flowers, tube length and nectar production) as covariates, and nutrient treatment (control vs. N or P supplementation) and the trait × nutrient treatment interactions as fixed factors. Statistical support for a trait × N (or P) addition interaction would support nutrient‐mediated selection. We quantified nutrient‐mediated selection (β_N‐C_ or β_P‐C_) for each trait by subtracting the selection estimate for plants receiving N or P addition (β_N_ or β_P_) from those of plants experiencing natural nutrient levels (β_C_).

## 3 Results

### 3.1 Effects of Nutrient Addition on Plant Traits, Seed Number, and Pollinator Visitation

Our nutrient‐addition treatments affected all traits except flower number, yet these effects differed among years (Table S1 and Figure S1). Plants subject to nutrient addition grew 5.8%–22.4% taller, except in the 2017 study of the +P treatment. Plants subject to nutrient addition had 10.7%–20.4% shorter tubes, except in the 2015 study of the +N treatment. In 2016, the nectar volume per flower decreased by 40.8% and 71.2% in the +N and +P treatments, respectively.

Nutrient addition also affected seed number per plant, yet in opposite directions between years (Table S1 and Figure S2). Plants subject to N addition produced 40.8% more seeds in 2015, but 31.7% fewer seeds in 2016. Plants subject to P addition produced 27.6% fewer seeds in 2016, but 38.3% more seeds in 2017. The opportunity for selection varied both among treatments and among years (Table S2).

The pollinator visitation rate varied among years but not among treatments, except for an increase in the +P treatment in 2017 (Table S1; Figure S2). In the control plots, pollinator visitation rates were correlated with plant height (2015, *r* = 0.499, *p* = 0.005; 2016, *r* = −0.45, *p* = 0.013) and flower number (*r* = 0.505, *p* = 0.004) in 2015. In the nutrient‐addition plots, however, linear regression analysis showed that pollinator visitation rates were not detectably related to plant height (N addition: both *r* < 0.1, *p* > 0.7; P addition: both *r* < 0.3, *p* > 0.2), flower number (N addition: both *r* < 0.3, *p* > 0.1; P addition: both *r* < 0.2, *p* > 0.15), and the other two traits (in all treatments: *r* < 0.2, *p* > 0.1).

### 3.2 Effects of Nutrient Addition on Phenotypic Selection

**FIGURE 1:**
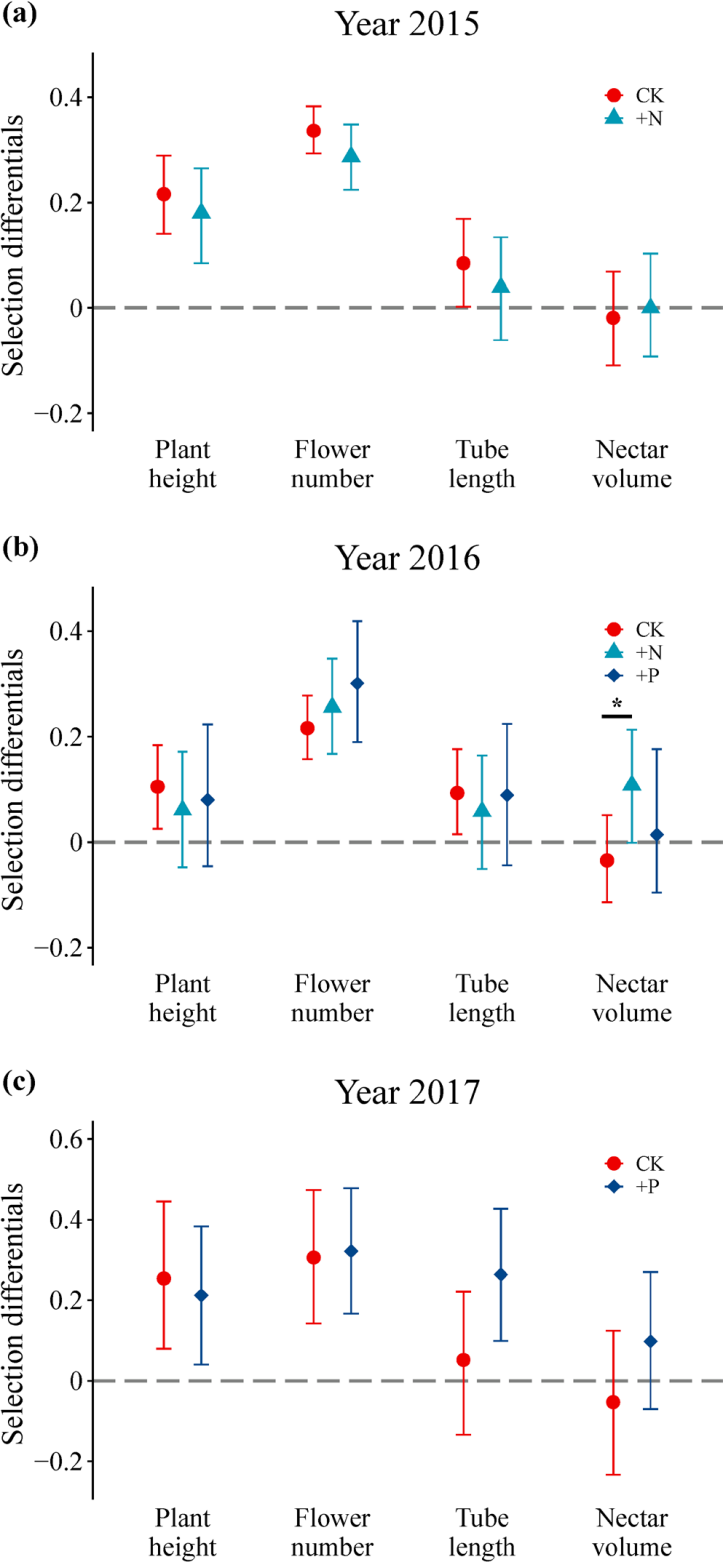
Selection differentials for floral and plant‐level traits of *Pedicularis szetschuanica* across experimental treatments. Treatments include control (C), P addition (+P), and N addition (+N). Asterisks (*) indicate a statistically detectable difference.

**FIGURE 2:**
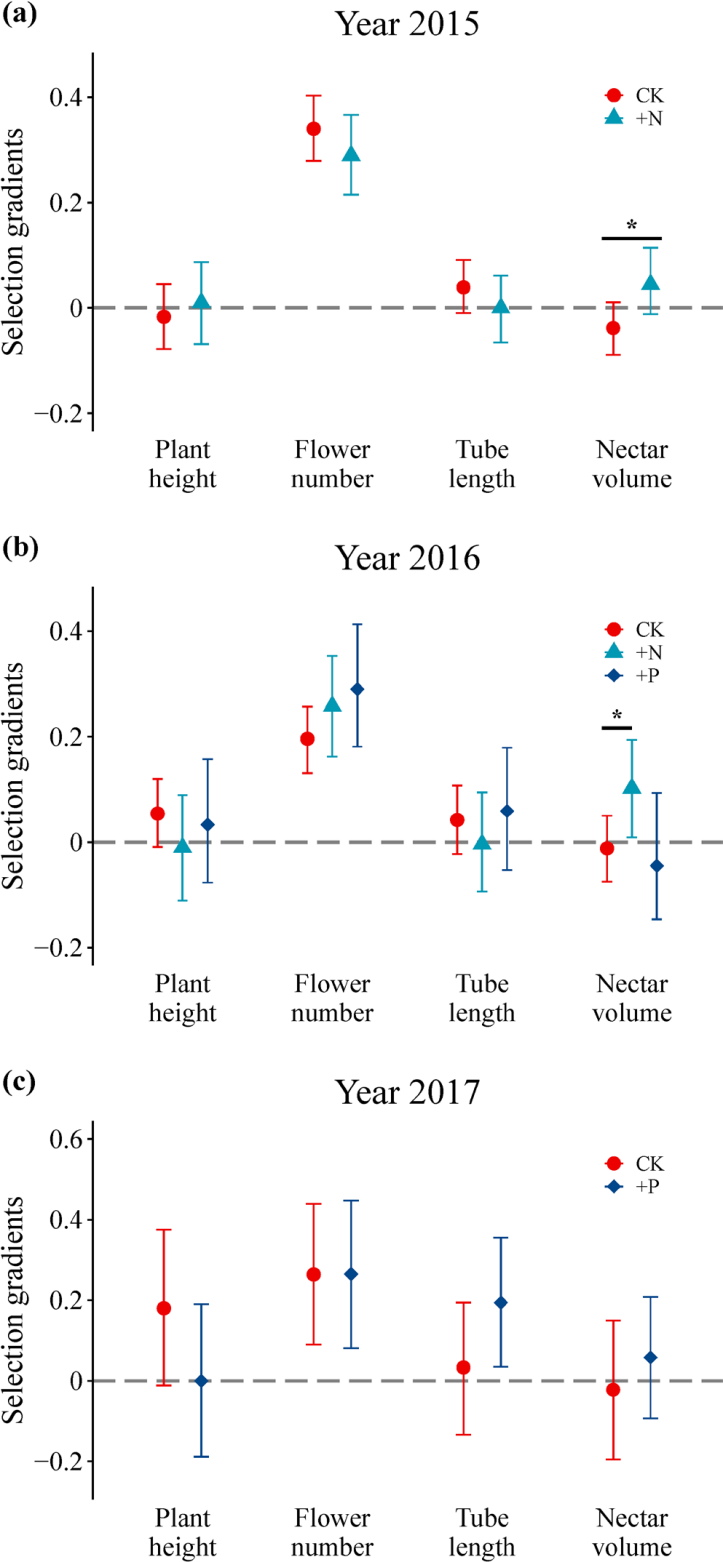
Selection gradients for floral and plant‐level traits of *Pedicularis szetschuanica* across experimental treatments. Treatments include control (C), P addition (+P), and N addition (+N). Asterisks (*) indicate a statistically detectable difference.

In the control treatment, we detected selection for taller plants with larger floral displays across all years (Figures 1, 2 and Tables S3, S4). Comparing selection differential (Figure 1) to multivariate (Figure 2) gradients revealed that selection usually acted directly on flower number and indirectly on plant height. The nitrogen‐addition treatment led to positive nitrogen‐mediated selection on nectar volume in both years (Figure 2). For the other traits, there was no effect of nutrient addition on selection.

Assessing patterns of selection and the opportunity for selection across traits and treatments (Figure S3) revealed a tendency for selection to be weak when the opportunity for selection was low, and both stronger on average and more variable when the opportunity for selection was higher.

## 4 Discussion

Variation in resource availability can influence patterns of natural selection on plant traits by altering phenotypic traits, mean fitness, the variance in fitness (opportunity for selection), and the functional relationships between traits and fitness. Our nutrient supplementation experiment demonstrates all these effects. Nutrient addition generally increased plant height and reduced tube length and nectar production per flower of *Pedicularis szetschuanica*. Surprisingly, the effects of N and P addition on seed number per plant were reversed between years, suggesting that variation in climate or other environmental factors influences the effect of nutrient addition. Despite variation in traits and fitness among nutrient treatments, patterns of selection changed only for nectar production, for which we detected positive N‐mediated selection. Selection on flower number and nectar production tended to be stronger when the opportunity for selection was higher.

### 4.1 Effects of Nutrient Addition on Plant Traits and Fitness

Our chosen *P. szetschuanica* phenotypic traits responded differently to N and P addition. Plant height increased as expected in response to nutrient addition, while we did not expect the observed reductions in flower size and nectar volume in response to nutrient addition. Greater plant height is a common response to nutrient addition (Dickson et al. 2014; Funk et al. 2017; Lin et al. 2020; Walter et al. 2017; Waterton et al. 2022; Cheng et al. 2023), and is thought to reflect at least in part the key role of light competition in plant communities (Hautier et al. 2009; Eskelinen et al. 2022). Tall grass species are an important component of the vegetation at our study site; thus, taller *P. szetschuanica* individuals may compete better for limiting light resources (Cheng et al. 2023).

The observed lack of change in flower number, along with the reduced flower size and nectar production in the nutrient‐addition treatments, was quite unexpected. This is because previous studies have reported increased flower number, larger flowers, and more nectar in response to experimental nutrient addition (Munoz et al. 2005; Burkle and Irwin 2009, 2010; Wu et al. 2022). We speculate that this discrepancy may reflect a resource‐allocation trade‐off in response to intensified light competition after nutrient addition. Plant competition is known to affect floral display size, flower production, and nectar quantity (Partzsch and Bachmann 2011; Flacher et al. 2015, 2017, 2020), and more so with more intense competition (Flacher et al. 2015, 2017). If so, our results could be explained by *P. szetschuanica* individuals in the nutrient addition plots investing more resources into vertical growth to improve light capture. As a result, fewer resources would be directed towards other attractive traits such as flower size and nectar production. Additionally, the reduced nectar production could be attributed to physical constraints of the flower organ as the flowers became smaller, while nectar production is highly plastic and can respond to a variety of environmental factors, including water availability, ambient humidity, and temperature (Parachnowitsch et al. 2019). Here, we did not quantify nectar sugar concentration and content, as these components can be influenced by abiotic factors and subsequently affect pollinator visitation and plant reproductive success (Parachnowitsch et al. 2019).

Seed number per plant both increased and decreased in response to nutrient addition, suggesting a complex and context‐dependent relationship between resource availability and plant fitness. Both N and P are important limiting nutrients for plant reproduction in terrestrial ecosystems, and numerous studies have demonstrated increased seed production in N and P enrichment experiments (e.g., Munoz et al. 2005; Caruso et al. 2005; Burkle and Irwin 2009, 2010; Bogdziewicz et al. 2017; Su et al. 2021, but see DiManno and Ostertag 2016). The reduced seed number of *P. szetschuanica* observed in the nutrient‐addition plots in 2016 may relate to stronger competition for light with taller grasses in the community. Because our focal alpine meadow community has a clear vertical structure, N addition reduced the number of short species but increased the number of tall species (Cheng et al. 2023). Indeed, after nutrient addition, the *P. szetschuanica* population in the alpine meadow community is currently declining with local extinction of individuals in some of the plots in 2019 (i.e., after 8 years of nutrient addition, personal observation by Zhao Zhigang). Alternatively, interannual climate variability could drive seed production fluctuations through its effects on pollinator communities and pollination efficacy (Inouye 2020), particularly given the pollen limitation common in alpine plants like *P. szetschuanica* (Wang et al. 2023). These contrasting mechanisms highlight the complex interplay between nutrient availability, competition, and pollination dynamics in shaping plant reproductive success.

### 4.2 Effects of Nutrient Addition on Phenotypic Selection

The substantial variation in mean fitness across years and treatments translated into the expected variation in the opportunity for selection (Figure S3 and Table S2). In environments where mean fitness is low, the variance in fitness and thus the opportunity for selection is usually high (Arnold and Wade 1984; Rundle and Vamosi 1996). Under these circumstances, we expect selection to be stronger on average and more variable across traits than when mean fitness is greater, i.e., lower opportunity for selection (Trunschke et al. 2017; Albertsen et al. 2021; Opedal 2021). Previous studies have shown that N addition can reduce the opportunity for selection in plant populations by increasing mean fitness (e.g., Mattila and Kuitunen 2000; Wu et al. 2023), yet others have found no change (Case and Ashman 2007; Sletvold et al. 2017; Waterton et al. 2022). In our study, the variable patterns across years led to substantial variation in the opportunity for selection, and we detected the expected increase in both the mean and variance in selection with increasing opportunity for selection, especially for flower number and nectar volume.

Despite substantial variation in plant traits and seed number across experimental nutrient treatments, we detected only a few cases of changes in patterns of phenotypic selection. Selection favored taller plants as expected, but this selection was indirect via correlated traits, notably flower number. Furthermore, nutrient addition did not alter patterns of selection on plant height despite the increase in individual height. This may be explained by the lack of effect of plant height on pollinator visitation to *P. szetschuanica* at the study site, or possibly that plant height was not favored through light competition. These observations suggest that, at this study site, resource addition may change plant height and fitness equally across individuals. In a different long‐term N addition experiment, Waterton et al. (2022) similarly failed to detect nitrogen‐mediated selection for plant height of the annual grass *Setaria faberi* despite greater light asymmetry. Selection favored increased flower number in *P. szetschuanica*, but this pattern was similar across all treatments and thus not influenced by N and P addition. It might be a simple result of flower production if *P. szetschuanica* individuals are not pollen‐limited. Similarly, resource supplementation did not affect phenotypic selection on floral traits in *Asclepias syriaca* , in which nutrient addition affected fruit production rather than trait distributions (Caruso et al. 2005), nor in the orchid *Dactylorhiza lapponica*, in which floral traits and fruit production remained similar after nutrient addition (Sletvold et al. 2017). On the other hand, resource‐mediated selection on floral traits may be related to pollination levels (Campbell and Bischoff 2013). For example, Wu et al. (2023) found that nutrient addition strengthened selection for smaller corolla size and more flowers in *Spiranthes sinensis* , but only when pollination was unreliable.

In the present study, nutrient addition primarily intensified selection for larger nectar volumes, leading to greater reproductive success for individuals producing more nectar. Nitrogen‐mediated selection for greater nectar production may have arisen from a change in the trait‐fitness relationship, as both the mean seed number per plant and the mean nectar production per flower decreased in the +N plots in 2016. Given that nectar is the main reward for pollinators in this system, we would usually expect selection on nectar volume to arise through pollinator foraging decisions (Parachnowitsch et al. 2019). However, the nectar volume of *P. szetschuanica* flowers did not affect the pollinator visitation rate at our study site, suggesting that other factors may be involved. Tong et al. (2019) reported significant variation in nectar production and sugar concentration among plants of a *Pedicularis* species, and they found that manipulation of nectar production can alter the foraging behavior of bumble‐bees. Therefore, both inter‐ and intra‐individual variation in nectar production likely influences pollinator foraging and consequently affects selection (Zhao et al. 2016).

Tube length was not under selection at our study site, despite the observed variation in tube length among treatments and years. Tube length is often involved in flower‐pollinator fit, and the lack of detectable selection again supports the hypothesis that pollinators play a limited role in selection in this system. Alternatively, limited variation of this trait reduces the possibility of detecting statistically significant phenotypic selection (Trunschke et al. 2019, 2020). In general, resource‐related changes in trait distributions may affect selection intensity also without changing the fitness surface (Weis et al. 1992; Steele et al. 2011), and further study is needed to understand the lack of such effects in *P. szetschuanica*.

## Author Contributions

**Lu Ningna:** writing – original draft (equal). **Hou Meng:** writing – original draft (equal). **Ma Yan:** writing – original draft (equal). **Øystein H. Opedal:** writing – original draft (equal). **Zhao Zhigang:** writing – original draft (equal).

## Conflicts of Interest

The authors declare no conflicts of interest.

## Supporting information


**Figure S1.** Effects of nutrient addition (N and P) on plant height and floral traits of *Pedicularis szetschuanica*. Linear mixed models were used, different letters represent statistically detectable differences (*p* < 0.05).

**Figure S2.** Effects of nutrient addition (N and P) on pollinator visitation rate and mean seed number of *Pedicularis szetschuanica* populations. Linear mixed models were used, different letters represent statistically detectable differences (*p* < 0.05).

**Figure S3.** Relationships between selection strength (absolute value of selection differentials and gradients) and opportunity for selection across treatments. Treatments include control (C), P addition (+P) and N addition (+N). The error bars represent standard errors.

**Table S1.** Effects of nutrient addition (N and P) and year, and their interaction on plant traits, mean visitation rate of pollinators, and seed number. Bold text indicates statistically significant estimates (< 0.05).
**Table S2.** Opportunity for selection (*I*) per year and treatment. Values in parentheses are 95% confidence intervals.
**Table S3.** Selection differentials (± SE) for plant height and floral traits in the nutrient addition treatments. (*β*_N‐C_ means N‐mediated selection, *β*_P‐C_, P‐mediated selection). Bold text indicates statistically significant estimates (< 0.05).
**Table S4.** Selection gradients (± SE) for plant height and floral traits in the nutrient addition treatments. (*β*_N‐C_ means N‐mediated selection, *β*_P‐C_, P‐mediated selection). Bold text indicates statistically significant estimates (< 0.05).

## Data Availability

The data that support the findings of this study are openly available in “figshare” at https://figshare.com/s/0647b89bcc7a4d9188b2.
