# Supplementary figures and images for: Nutrient Enrichment Alters Phenotypic Selection on Plant Traits in an Annual Herb on the Tibetan Plateau
Source: Ecol Evol. 2025 Jun 14;15(6):e71592. doi: 10.1002/ece3.71592 (PMC12166381; doi:10.1002/ece3.71592)

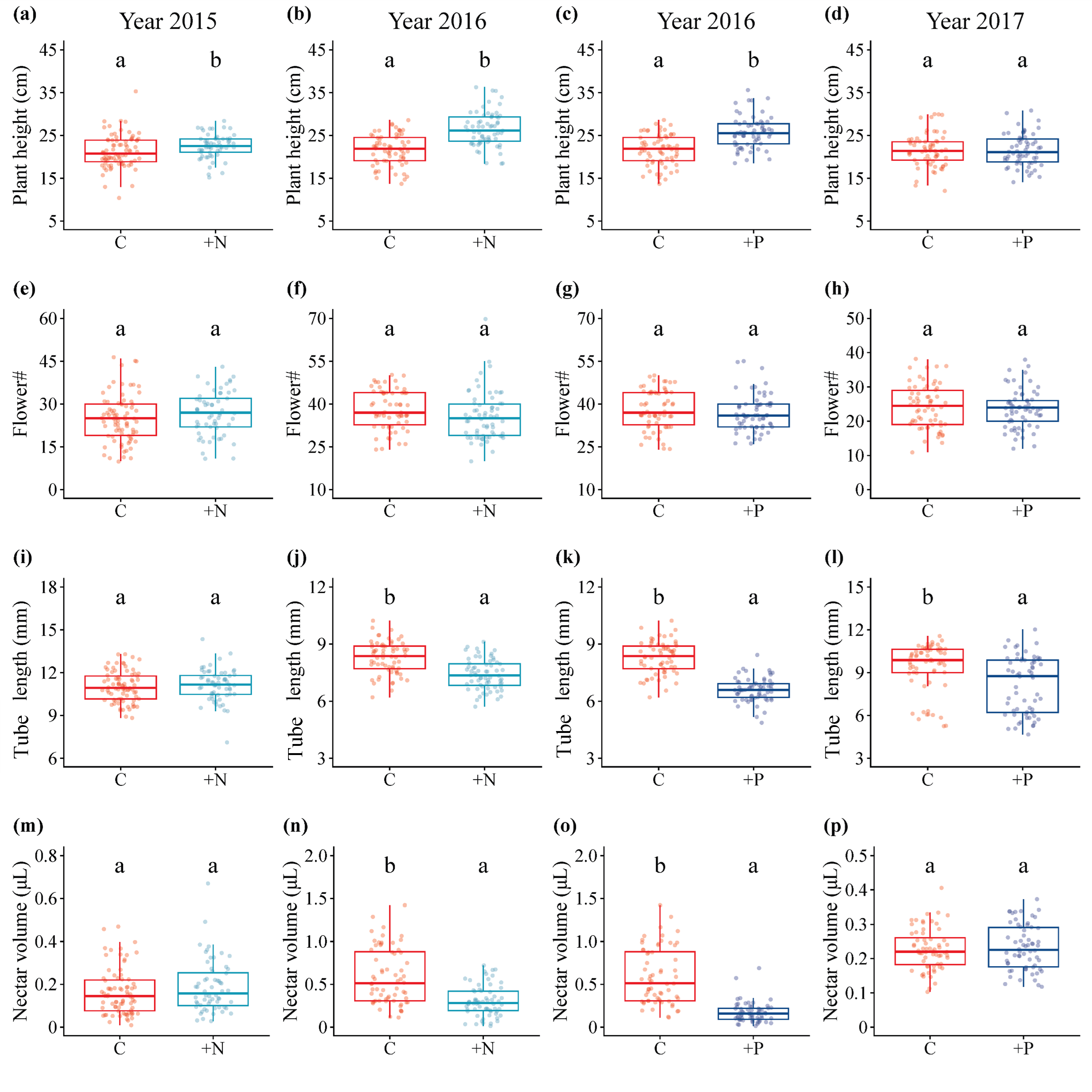

Supplement: Supplementary file 1 — Figure S1. Effects of nutrient addition (N and P) on plant height and floral traits of Pedicularis szetschuanica. Linear mixed models were used, different letters represent statistically detectable differences (p < 0.05). [file ECE3-15-e71592-s004.tif]

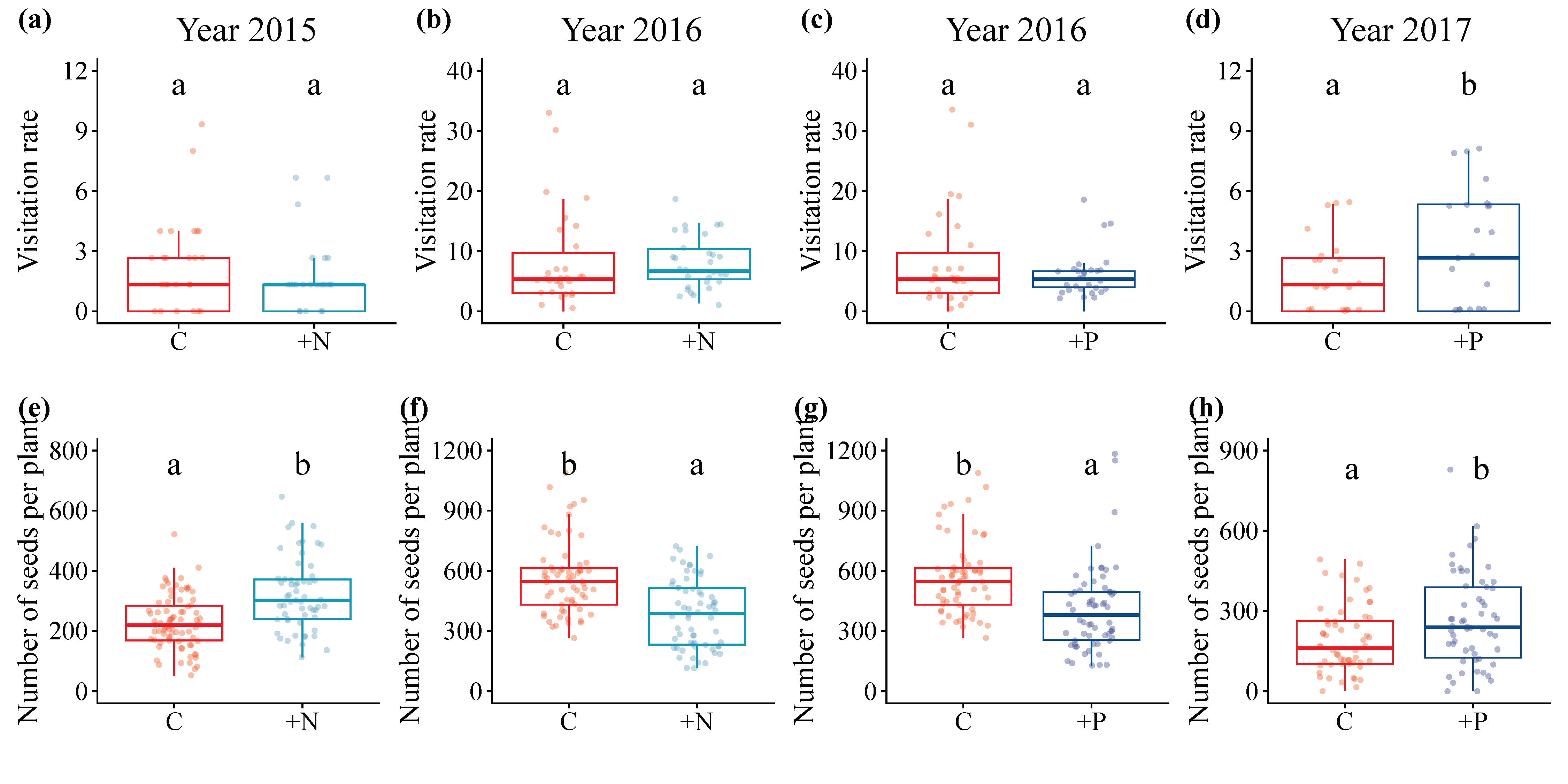

Supplement: Supplementary file 2 — Figure S2. Effects of nutrient addition (N and P) on pollinator visitation rate and mean seed number of Pedicularis szetschuanica populations. Linear mixed models were used, different letters represent statistically detectable differences (p < 0.05). [file ECE3-15-e71592-s001.tif]

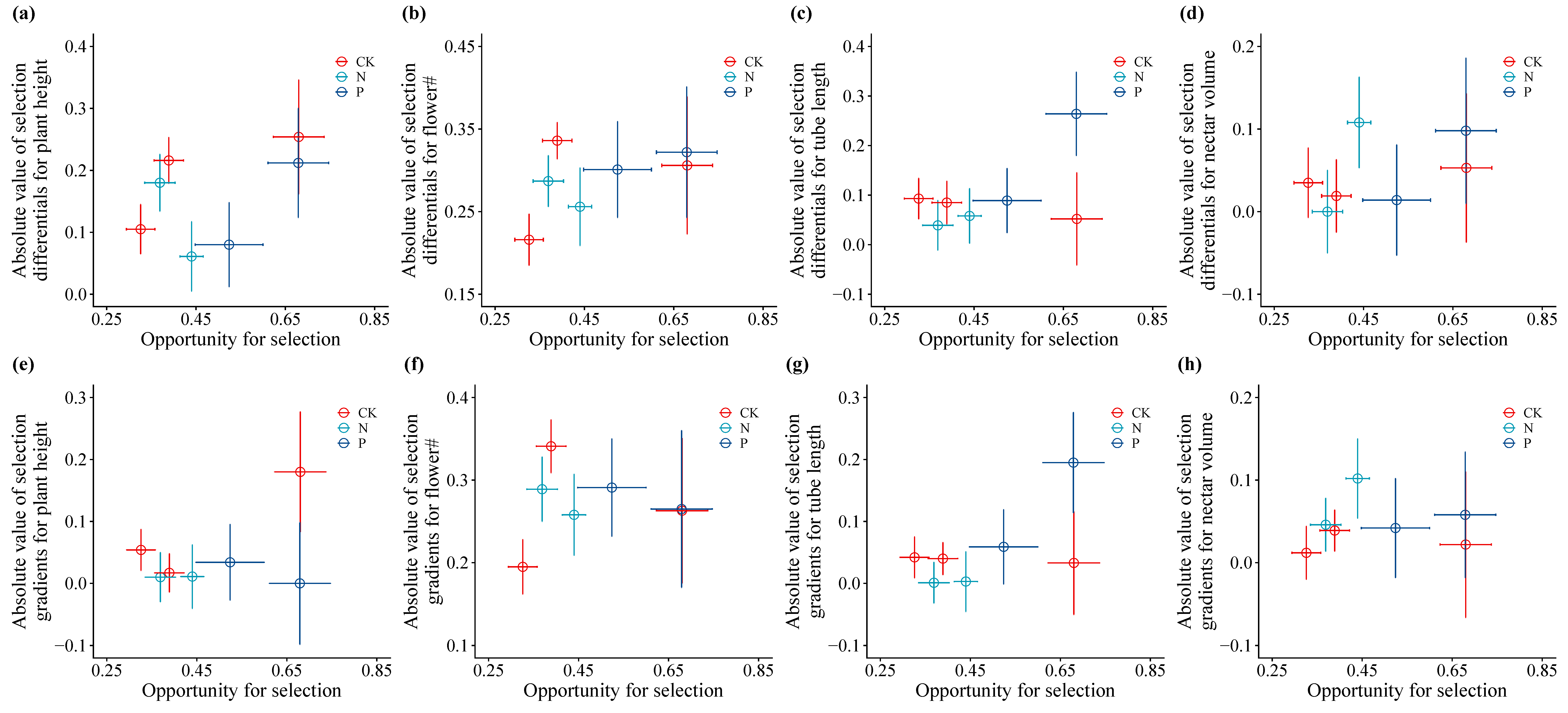

Supplement: Supplementary file 3 — Figure S3. Relationships between selection strength (absolute value of selection differentials and gradients) and opportunity for selection across treatments. Treatments include control (C), P addition (+P) and N addition (+N). The error bars represent standard errors. [file ECE3-15-e71592-s003.tif]
