# Supplementary material for: Nutrient Enrichment Alters Phenotypic Selection on Plant Traits in an Annual Herb on the Tibetan Plateau
Source: Ecol Evol. 2025 Jun 14;15(6):e71592. doi: 10.1002/ece3.71592 (PMC12166381; doi:10.1002/ece3.71592)
Supplement: Supplementary file 4 — Table S1. Effects of nutrient addition (N and P) and year, and their interaction on plant traits, mean visitation rate of pollinators, and seed number. Bold text indicates statistically significant estimates (< 0.05). Table S2. Opportunity for selection (I) per year and treatment. Values in parentheses are 95% confidence intervals. Table S3. Selection differentials (± SE) for plant height and floral traits in the nutrient addition treatments. (β N‐C means N‐mediated selection, β P‐C, P‐mediated selection). Bold text indicates statistically significant estimates (< 0.05). Table S4. Selection gradients (± SE) for plant height and floral traits in the nutrient addition treatments. (β N‐C means N‐mediated selection, β P‐C, P‐mediated selection). Bold text indicates statistically significant estimates (< 0.05). [file ECE3-15-e71592-s002.docx]

**Table S1.** Effects of nutrient addition (N and P) and year and their interaction on plant traits, mean visitation rate of pollinators, and seed number. Bold text indicates statistically significant estimates (<0.05).

|  | *df* | Plant height (cm) | |  | Flower# | |  | Tube length (mm) | |  | Nectar volume (ml) | |  | Visitation rate (visits/h) | |  | Seed number per plant | |
| --- | --- | --- | --- | --- | --- | --- | --- | --- | --- | --- | --- | --- | --- | --- | --- | --- | --- | --- |
|  |  | *F* value | *P* |  | *F* value | *P* |  | *F* value | *P* |  | *F* value | *P* |  | *F* value | *P* |  | *F* value | *P* |
| N addition | 1 | **43.759** | **<0.001** |  | 0.068 | 0.795 |  | **6.030** | **0.014** |  | **40.123** | **<0.001** |  | 0.325 | 0.569 |  | **3.900** | **0.049** |
| P addition | 1 | **20.240** | **<0.001** |  | 1.105 | 0.294 |  | **88.351** | **<0.001** |  | **106.789** | **<0.001** |  | 0.900 | 0.344 |  | **5.145** | **0.024** |
| Year | 2 | **24.451** | **<0.001** |  | **150.355** | **<0.001** |  | **244.190** | **<0.001** |  | **78.212** | **<0.001** |  | **41.881** | **<0.001** |  | **92.134** | **<0.001** |
| N × Year | 1 | **16.059** | **<0.001** |  | **5.446** | **0.020** |  | **10.381** | **0.001** |  | **61.508** | **<0.001** |  | 0.052 | 0.820 |  | **47.853** | **<0.001** |
| P × Year | 1 | **19.284** | **<0.001** |  | 0.120 | 0.729 |  | 1.955 | 0.163 |  | **110.585** | **<0.001** |  | **5.187** | **0.024** |  | **31.371** | **<0.001** |

**Table S2.** Opportunity for selection (*I*) per year and treatment. Values in parentheses are 95% confidence intervals.

| Year | *I*_C_ | *I*_N_ | *I*_P_ |
| --- | --- | --- | --- |
| 2015 | 0.389 (0.339, 0.482) | 0.369 (0.312, 0.463) |  |
| 2016 | 0.326 (0.276, 0.401) | 0.440 (0.396, 0.502) | 0.524 (0.410, 0.725) |
| 2017 | 0.680 (0.581, 0.809) |  | 0.679 (0.578, 0.863) |

**Table S3.** Selection differentials (± SE) for plant height and floral traits in the nutrient addition treatments. (*β*_N-C_ means N-mediated selection, *β*_P-C_, P-mediated selection). Bold text indicates statistically significant estimates (<0.05).

|  | Traits | *β*_C_ | *β*_N_ | *β*_P_ | *β*_N-C_ | *β*_P-C_ |
| --- | --- | --- | --- | --- | --- | --- |
| (a) Year 2015 | | | | | | |
|  | Plant height (cm) | **0.216 ± 0.037** | **0.180 ± 0.046** |  | -0.037 ± 0.059 |  |
|  | Flower# | **0.336 ± 0.022** | **0.287 ± 0.031** |  | -0.050 ± 0.037 |  |
|  | Tube length (mm) | **0.085 ± 0.043** | 0.039 ± 0.050 |  | -0.046 ± 0.066 |  |
|  | Nectar volume (ul) | -0.019 ± 0.044 | 0.000 ± 0.050 |  | 0.019 ± 0.067 |  |
| (b) Year 2016 | | | | | | |
|  | Plant height (cm) | **0.105 ± 0.040** | 0.061± 0.056 | 0.080 ± 0.068 | -0.044 ± 0.069 | -0.025 ± 0.079 |
|  | Flower# | **0.216 ± 0.031** | **0.256 ± 0.047** | **0.301 ± 0.058** | 0.040 ± 0.055 | 0.085 ± 0.064 |
|  | Tube length (mm) | **0.093 ± 0.041** | 0.058 ± 0. 055 | 0.089 ± 0.065 | -0.035 ± 0.069 | -0.003 ± 0.078 |
|  | Nectar volume (ul) | -0.035 ± 0.042 | **0.108 ± 0.055** | -0.014 ± 0.067 | **0.143 ± 0.069** | 0.049 ± 0.079 |
| (c) Year 2017 | | | | | | |
|  | Plant height (cm) | **0.254 ± 0.092** |  | **0.212 ± 0.088** |  | -0.042 ± 0.128 |
|  | Flower# | **0.306 ± 0.083** |  | **0.322 ± 0.079** |  | 0.016 ± 0.115 |
|  | Tube length (mm) | 0.052 ± 0.093 |  | **0.264 ± 0.084** |  | 0.212 ± 0.124 |
|  | Nectar volume (ul) | -0.053 ± 0.090 |  | 0.098 ± 0.088 |  | 0.152 ± 0.125 |

**Table S4.** Selection gradients (± SE) for plant height and floral traits in the nutrient addition treatments. (*β*_N-C_ means N-mediated selection, *β*_P-C_, P-mediated selection). Bold text indicates statistically significant estimates (<0.05).

|  | Traits | *β*_C_ | *β*_N_ | *β*_P_ | *β*_N-C_ | *β*_P-C_ |
| --- | --- | --- | --- | --- | --- | --- |
| (a) Year 2015 | | | | | | |
|  | Plant height (cm) | -0.017 ± 0.031 | 0.010 ± 0.040 |  | 0.028 ± 0.050 |  |
|  | Flower# | **0.341 ± 0.032** | **0.289 ± 0.039** |  | -0.052 ± 0.049 |  |
|  | Tube length (mm) | 0.040 ± 0.026 | -0.001 ± 0.033 |  | -0.040 ± 0.040 |  |
|  | Nectar volume (ul) | -0.039 ± 0.025 | 0.046 ± 0.032 |  | **0.085 ± 0.040** |  |
| (b) Year 2016 | | | | | | |
|  | Plant height (cm) | 0.054 ± 0.033 | -0.011 ± 0.051 | 0.034 ± 0.061 | -0.065 ± 0.060 | -0.020 ± 0.068 |
|  | Flower# | **0.195 ± 0.033** | **0.258 ± 0.049** | **0.291 ± 0.059** | 0.063 ± 0.058 | 0.096 ± 0.067 |
|  | Tube length (mm) | 0.042 ± 0.033 | -0.003 ± 0.048 | 0.059 ± 0.060 | -0.045 ± 0.059 | 0.017 ± 0.069 |
|  | Nectar volume (ul) | -0.012 ± 0.032 | **0.102 ± 0.048** | -0.042 ± 0.060 | **0.114 ± 0.056** | -0.030 ± 0.069 |
| (c) Year 2017 | | | | | | |
|  | Plant height (cm) | 0.180 ± 0.097 |  | 0.000 ± 0.098 |  | -0.180 ± 0.139 |
|  | Flower# | **0.263 ± 0.088** |  | **0.265 ± 0.095** |  | 0.001 ± 0.129 |
|  | Tube length (mm) | 0.033 ± 0.083 |  | **0.195 ± 0.081** |  | 0.162 ± 0.116 |
|  | Nectar volume (ul) | -0.022 ± 0.088 |  | 0.058 ± 0.076 |  | 0.080 ± 0.118 |
